# Clinicopathological characteristics and prognosis of gastrointestinal vascular tumours

**DOI:** 10.1038/s41598-021-94821-1

**Published:** 2021-08-09

**Authors:** Zhou Haibin, Wang Lingling, Zhang Lexing, Bao Xumin, Wang Yingyu, Yang Jianfeng, Zhang Xiaofeng

**Affiliations:** 1 Department of Gastroenterology, Key Laboratory of Clinical Cancer Pharmacology and Toxicology Research of Zhejiang Province, Affiliated Hangzhou First People’s Hospital, Zhejiang University School of Medicine, No. 261, Huansha Road, Hangzhou, China; 2 Department of Pathology, Affiliated Hangzhou First People’s Hospital, Zhejiang University School of Medicine, Hangzhou, China; 3 Department of Radiology, Affiliated Hangzhou First People’s Hospital, Zhejiang University School of Medicine, Hangzhou, China; 4 Department of Gastroenterology, Affiliated Jiaxing City First People’s Hospital, Jiaxing, China

**Keywords:** Colorectal cancer, Gastrointestinal bleeding, Intestinal diseases, Colonoscopy, Oesophagogastroscopy

## Abstract

To evaluate the clinicopathological characteristics and prognosis of gastrointestinal vascular tumours. By reviewing the information from the electronic medical record system and pathology database of Hangzhou First People's Hospital affiliated with Zhejiang University School of Medicine and Jiaxing First People’s Hospital from June 2008 to December 2019, 31 patients pathologically diagnosed with vascular tumours were included in this study. The age of onset, sex differences, clinical manifestations, imaging and endoscopic characteristic manifestations, pathological characteristics, treatment methods and prognosis were analysed. The pathological classification was haemangiolymphangioma, haemangioma, and lymphangioma in 8, 14, and 9 cases, respectively. The age of onset was 44–66 years, with no significant difference according to sex (P = 0.583); 32.26% (10/31) of patients had no noticeable symptoms, 37.5% (12/31) of patients had gastrointestinal bleeding, and 6.45% (2/31) of patients, all with lymphangioma, had intestinal obstruction. The lesions were located in and below the duodenum. Endoscopy showed colour differences. Both endoscopic and surgical treatments were safe and effective. The mean survival time was 57.06 ± 35.64 months. Regarding vascular tumours without typical symptoms, the main pathological classification is haemangioma. Vascular tumours are often clinically identified because of bleeding or obstruction and can be treated with endoscopy or surgery. Clinical follow-up is recommended because no invasive manifestations or instances of recurrence were observed.

## Introduction

Vascular tumours (VTs) are rare, with an incidence rate of 0.12% to 0.28% among all digestive diseases^1^, and are a class of benign tumours originating in mesenchymal tissue. The pathological types include haemangiolymphangioma (HL), haemangioma (H) and lymphangioma (L), which are common in children and adolescents^2^ and rare in adults; multiple VTs can occur in the head, neck and limbs^3^, but VTs in the digestive tract are rare. A few cases are classified as "intussusception"^4^ or "anaemia of unknown cause"^5^. These reports and lack of relevant systematic research, because of the lack of understanding of this disease, have led to frequent misdiagnosis of the disease and unreasonable treatment plans. To clarify the clinical characteristics of vascular tumours and provide the basis for clinical diagnosis and treatment, this study retrospectively analysed the clinical data of 31 patients with VTs diagnosed at the Hangzhou First People’s Hospital affiliated with the Zhejiang University School of Medicine and First People's Hospital of Jiaxing City from June 2008 to December 2019, as reported below.

## Materials and methods

### Research subjects

Thirty-one patients diagnosed with VTs from June 2008 to December 2019 at the Hangzhou First People's Hospital affiliated with the Zhejiang University School of Medicine and Jiaxing First People's Hospital were divided into 3 groups according to pathology, as follows: haemangiolymphangioma group (HL group; 8 cases); haemangioma group (H group; 14 cases); lymphangioma group (L group; 9 cases). Differences in sex, clinical manifestations, characteristic imaging and endoscopy findings, surgical pathological characteristics, treatment methods and prognosis were examined.

### Research methods

#### Ethics

All the methods and data analyses were approved by the local ethics board of Hangzhou First People's Hospital, Zhejiang University School of Medicine.

#### Data

By reviewing the information from the electronic medical record system and pathology database of Hangzhou First People’s Hospital affiliated with Zhejiang University School of Medicine and First People's Hospital of Jiaxing City from June 2008 to December 2019, 31 patients pathologically diagnosed with vascular tumours were included in the present study.

#### Measurement

Olympus endoscopy and Fujifilm endoscopy were used in gastroscopy or colonoscopy for all patients, and various models were randomly used, such as CF-HQ290I (Olympus colonoscopy), GIF-H290Z (Olympus gastroscopy) and EC-760R-V/M (Fujifilm colonoscopy). Additionally, a blood cell analyser and an automated biochemical analyser were used. All the available slides (haematoxylin and eosin stains, PAS, and Masson trichrome) were subjected to immunohistochemistry.

#### Pathological definitions

A haemangioma is a benign tumour comprising capillary-like blood vessels of small or large calibre. It comprises thin-walled blood-filled vessels lined by a single layer of flat cytologically banal endothelial cells. Lymphangioma is formed by the accumulation and proliferation of lymphangioma. There may be lymph or lymphocytes in the lumen. A haemangiolymphangioma is a mixed benign tumour formed by irregular hyperplasia of blood vessels and lymphatic vessels. It contains both haemangioma and lymphangioma, the proportion of which are different (From the 2013 WHO classification of bone and soft tissue tumours).

#### Statistical analysis

SPSS 22.0 statistical software was used for data analysis. Normally distributed continuous measurement data were expressed as means ± SD. Comparisons between groups were performed by one-way ANOVA. The least significant difference was used to compare multiple groups, and count data were expressed as the number of cases or rate (%). Comparisons between groups were performed by χ^2^. Fisher’s exact probability method was used to test for associations. Under different statistical methods, t, F and χ^2^ were used to display the variance, and P < 0.05 was considered statistically significant.

#### Type of study

The clinical data of the patients who had undergone endoscopy at the Gastroenterology of Hangzhou First People's Hospital affiliated with the Zhejiang University School of Medicine and First People’s Hospital of Jiaxing City from June 2008 to December 2019 were analysed retrospectively. Thirty-one patients pathologically diagnosed with vascular tumours in this period were analysed.

#### Statements concerning examinations and treatments

All the methods were performed in accordance with relevant guidelines and regulations. Written informed consent was obtained from all the subjects/LARs for this retrospective study.

## Results

### Age of onset

The age range was 19–84 years, with an average age of 52.94 ± 18.18 years. No statistically significant difference was found in the age of onset between the male and female patients (52.73 ± 17.42 vs. 53.13 ± 19.43, respectively; P = 0.583), with the age of onset ranging from 44 to 66 years (48.39%, 15/31).

According to the pathological type, the average ages in the HL, H, and L groups were 56 ± 13.26, 53.36 ± 20.92, 49.56 ± 18.75 years, respectively, and no significant difference was found among these three groups (F = 0.026; P = 0.773).

### Sex differences

Among the 31 patients, 15 were male (48%). The ratio of male to female patients by pathological type was as follows (male/female): HL group, 2/6; H group, 7/7; L group, 6/3.

### Clinical manifestations

Concerning the clinical manifestations, 32% (10/31) of the patients had no noticeable symptoms and were identified during a routine gastrointestinal examination; 23% (7/31) exhibited mild abdominal discomfort, such as mild abdominal pain and increased defecation frequency with occasional abdominal discomfort; 39% (12/31) exhibited signs of gastrointestinal bleeding, such as black stool, bloody stool, and dizziness and fatigue due to blood loss; 6% (2/31) exhibited signs of gastrointestinal obstruction, such as abdominal pain, nausea and vomiting. In addition to mild abdominal discomfort in the L group, 75% (3/4) of patients showed an increased defecation frequency, and no other pathological types were found in these patients. Symptoms of obstruction were found only in the L group, and the symptoms were typical signs of intestinal obstruction.

The symptoms (no noticeable symptoms/mild abdominal discomfort/manifestations of gastrointestinal bleeding/gastrointestinal obstruction) are summarized by the number of cases according to the pathological classification, as follows: HL group, 3/2/3/0; H group, 4/1/9/0; and L group, 3/4/0/2.

### Site of disease

**Figure 1:**
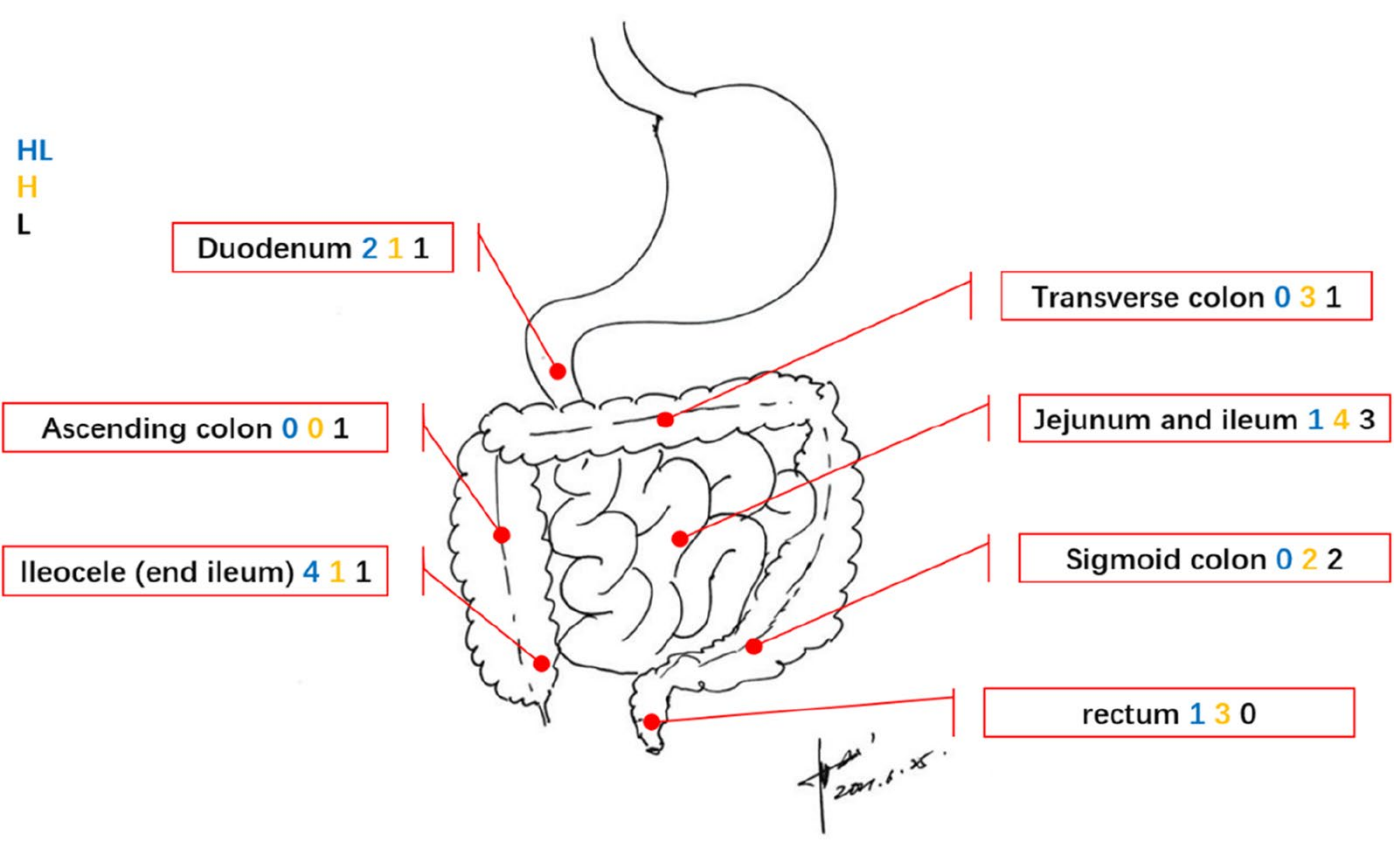
Analysis of various types of gastrointestinal vascular tumors (VTs) by site. All VTs were found in or below the duodenum; 58.06% (18/31) of lesions were in the small intestine, and 41.94% (13/31) of lesions were in the large intestine. There was no significant difference in the distribution of each pathological type, (Font color corresponds to type).

All VTs were found in or below the duodenum; 58% (18/31) of lesions were in the small intestine, and 42% (13/31) of lesions were in the large intestine. No significant difference was found in the distribution of each pathological type (Fig. 1).

### Characteristics of blood indicators

The haemoglobin level was significantly higher in the L group than in the HL and H groups (P < 0.05; Table 1). No significant difference was found in the levels of white blood cells, neutrophils, platelets, or C-reactive protein among the three groups (P > 0.05), and the carcinoembryonic antigen (CEA) and alpha-fetoprotein (AFP) levels were in the normal range.

**Table 1:** Analysis of blood indexes in gastrointestinal VTs patients (N = 31).

	HL (N = 8)	H (N = 14)	L (N = 9)	F	P
Age (range), years	56 ± 13.26 (30–77)	53.36 ± 20.92 (19–84)	49.56 ± 18.75 (22–71)	0.026	0.773
**Sex**					
Male, No	2	7	6		
Female, No	6	7	3		
WBC (10^9^/L)	5.74 ± 1.66	7.16 ± 2.95	6.71 ± 2.19	0.782	0.469
N%	66.11 ± 10.91	70.79 ± 15.2	69.11 ± 7.9	3.189	0.059
HB (g/L)	124.14 ± 23.88	87.3 ± 36.03	142.57 ± 15.61	4.317	0.025*
PLT (10^9^/L)	197.86 ± 48.19	172.7 ± 70.33	238.57 ± 84.81	2.659	0.091
CRP (mg/L)	2.79 ± 3.03	7.6 ± 8.46	10.71 ± 17.67	0.227	0.799
CEA (μg/L)	1.72 ± 1.27	2.5 ± 1.81	3.53 ± 1.65		
AFP (μg/L)	3.18 ± 1.62	2.7 ± 0.5	5.4 ± 3.96		

*L is significantly different from HL and H.

### Morphological characteristics (shape, colour, and size) of the lesion

**Figure 2:**
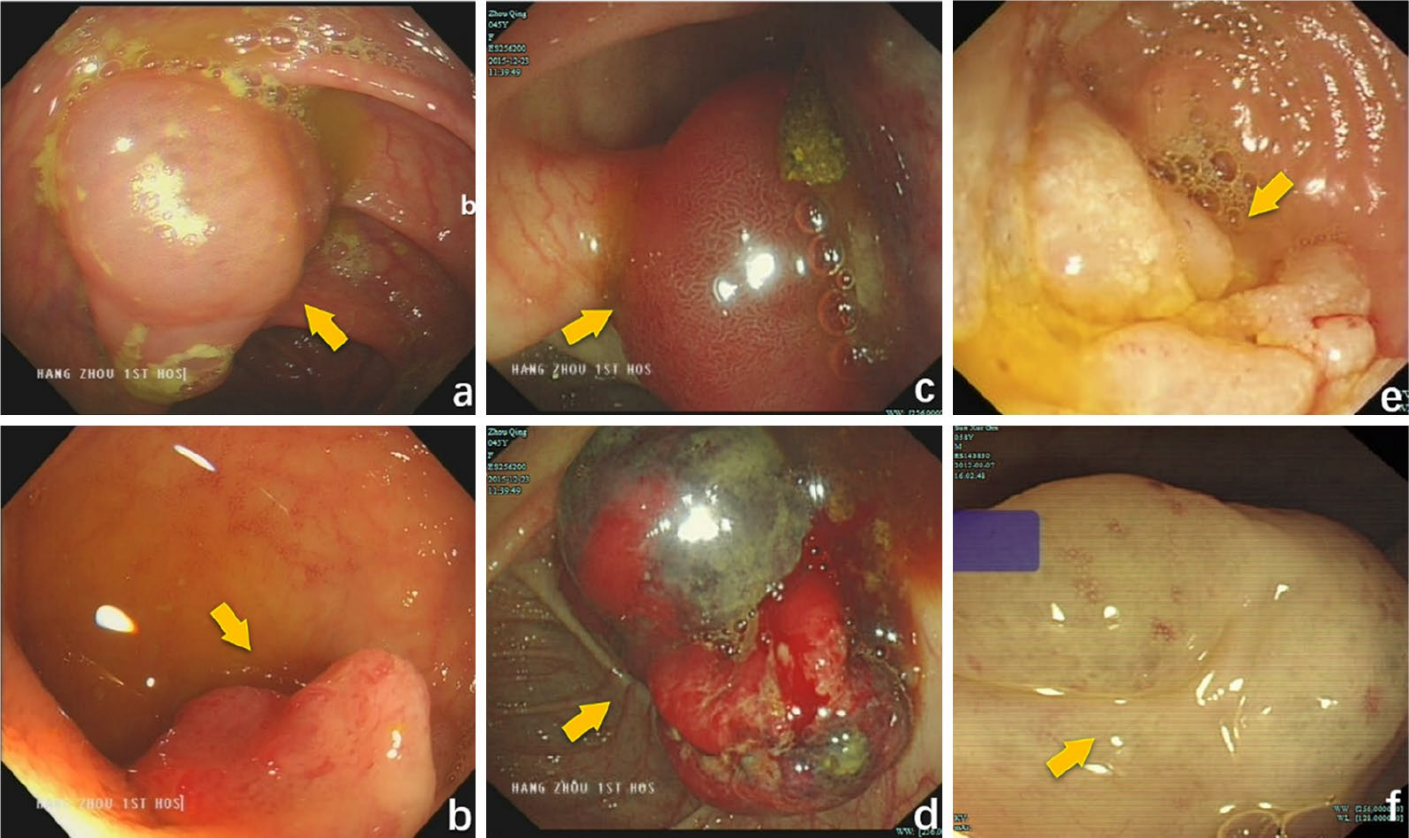
Analysis of various types of gastrointestinal VTs by endoscopic pictures, which correspond to those of Fig. 5. (**a)** Hemangiolymphangioma (HL) in the ileocecal region; b: HL in the terminal ileum; (**c**) Haemangioma (H) in the ileocecal region, H were mainly red, with the adventitia showing signs of blood filling; (**d**) H in the ileocecal region showing attached feces bleeding after flushing with normal saline; (**e**) Lymphangioma (L) in the ileocecal region; L were milky white, mucosal bulges with lateral changes; (**f**) Sigmoid lymphangioma; L presents an obvious cyst-like structure, containing lymph fluid.

**Figure 3:**
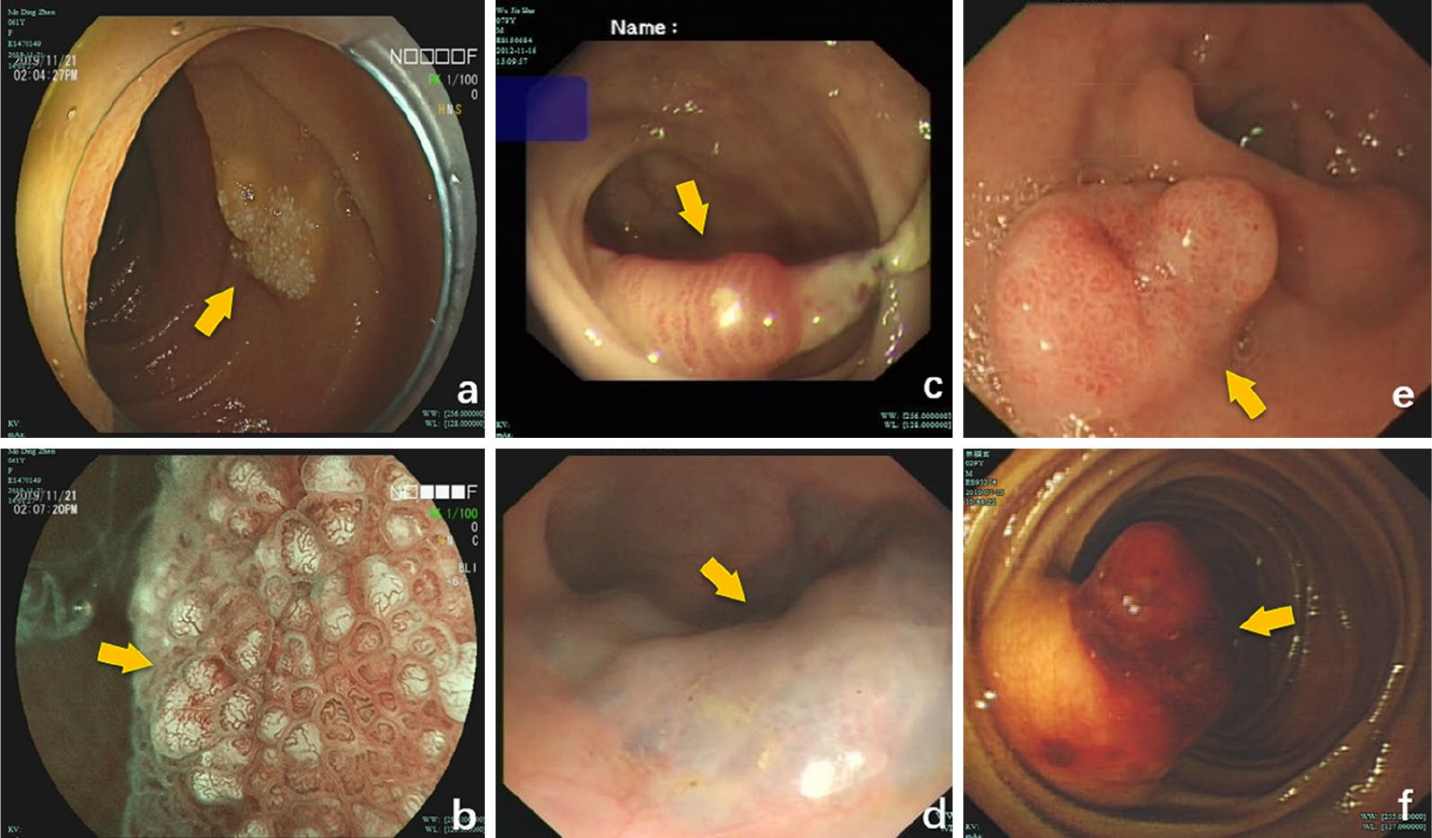
Supplementary endoscopic pictures. (**a**) Duodenal HL mimicking a lymphangioma, white spots with flaky changes can be observed by increasing the magnification of white granules, see enlarged view; (**b**) Duodenal HL, along with thick heterogeneous blood vessels and white opaque substances, indicating a mixture of lymphatic and vascular components. (**a**, magnifying endoscope); (**c**) transverse colonic H showing striations; H sometimes has such red stripes; (**d**) H in the ileocecal region, H sometimes has such dark red color; dark red areas similar to signs of enveloping venous blood; (**e**) Duodenal H; Lack of obvious capsule structure, but ulcer surface, the clinical manifestation of this case is bleeding: (**f**) small intestinal hemangioma showing bleeding.

The average size of all the tumours was 16.71 ± 12.1 mm. The size of the tumours in the HL group (17.5 ± 9.89 mm), H group (16.93 ± 12.39 mm), and L group (15.67 ± 14.57 mm) was not significantly different (P > 0.05) (F = 0.049, P = 0.952). Morphologically, H mainly developed laterally and was hemispherical (Fig. 2c), while HL and L showed no noticeable morphological characteristics and lateral development and globularity were dominant (Fig. 2a,b). HL was redder and whiter in colour, H was bright red and dark red (Fig. 3c,d), and L was milky white (see Fig. 2e,f; Table 2). More details are shown in the endoscopic images (Figs. 2 and 3).

**Table 2:** Morphological analysis of gastrointestinal VTs (N = 31).

	HL (N = 8)	H (N = 14)	L (N = 9)	F	P
**Shape**					
Flake	1	0	2		
Lateral development	3	3	3		
Hemisphere	4	8	2		
spherical	0	3	2		
**Colour**					
Dark red	1	2	0		
Red	3	12	0		
Pink	1	0	0		
Milky	3	0	9		
Size (mm)	17.5 ± 9.89	16.93 ± 12.39	15.67 ± 14.57	0.049	0.952

### Endoscopic and imaging features

**Figure 4:**
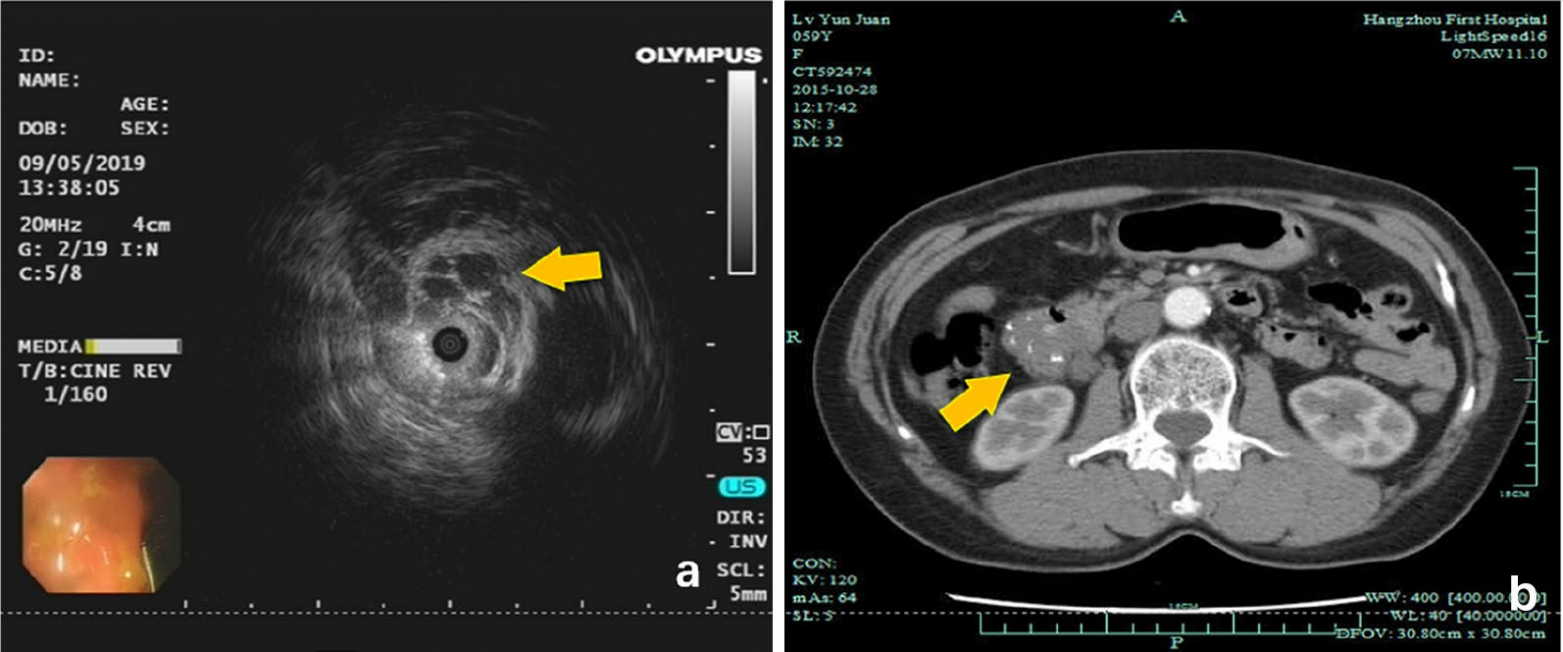
Supplement endoscopic pictures, in order to show imaging. **a**: HL of the ileocecal vasculature, Endoscopic ultrasound Endoscopic Ultrasound (EUS) showed that the hypoechoic shadows originated from the mucosal muscle layer and the submucosal layer, revealing an oval shape and internally separated (Fig. 2a, EUS); (**b**) Duodenum HL, Computed tomography (CT) of an angioma showed a low-density mass in the lower part of the duodenum, with multiple speckle-like high-density shadows; the size of the lesion was approximately 4.4 × 2.7 cm. Contrast-enhanced CT showed continuous uneven enhancement.

White light images (WLIs) were evaluated. On endoscopy, HL showed characteristics of H and L and was difficult to distinguish. HL exhibited a complete adventitia or was thin like H (Fig. 2a,b). Additionally, white spots with flaky changes were observed by increasing the magnification of white granules (Fig. 3a), along with thick heterogeneous blood vessels and white opaque substances (Fig. 3b), indicating a mixture of lymphatic and vascular components. Endoscopic ultrasound (EUS) showed that the hypoechoic shadows originated from the mucosal muscle layer and submucosal layer, revealing an oval shape and internal separation (Fig. 4a). H lesions were mainly red, with the adventitia showing signs of blood filling (Fig. 2c). The adventitia was also thin and easily bled after flushing (Fig. 2d), with striations (Fig. 3c) and dark red areas indicating enveloping venous blood (Fig. 3d) and a lack of a noticeable capsule structure but the presence of an ulcer surface. The clinical manifestation of this case was bleeding. (Fig. 3e); The L lesions were milky white, and mucosal bulges showed lateral changes (Fig. 2e) or noticeable capsules (Fig. 2f). Noticeable H bleeding could be observed under the endoscope (Fig. 3f). Computed tomography (CT) of an angioma showed a low-density mass in the lower part of the duodenum, with multiple speckle-like high-density shadows; the size of the lesion was approximately 4.4 × 2.7 cm. Contrast-enhanced CT showed continuous uneven enhancement (Fig. 4b).

### Characteristic pathological manifestations

**Figure 5:**
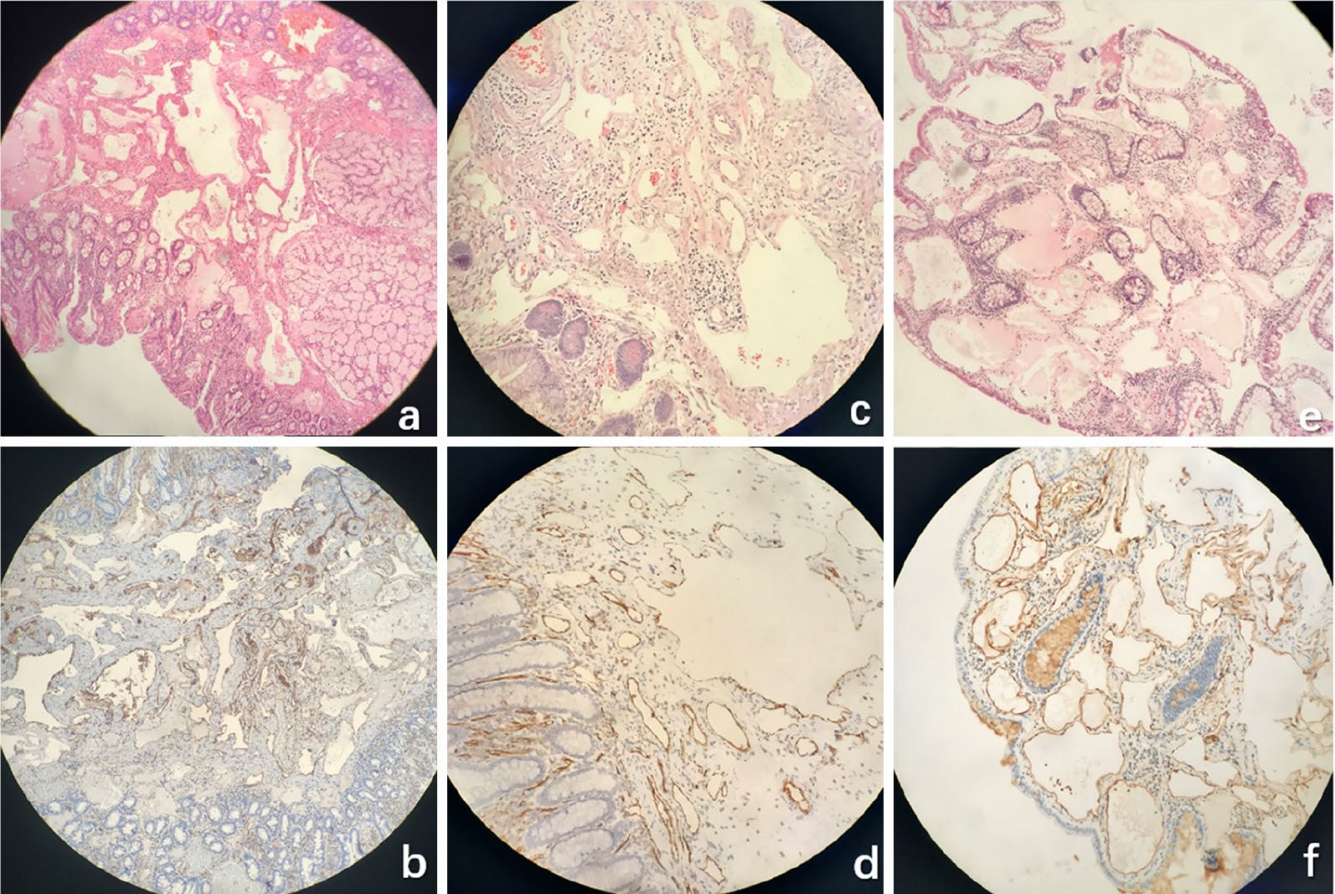
Analysis of various types of gastrointestinal VTs by pathological manifestations. (**a**) HL, HE × 200; (**b**) HL, IHC: CD34+/D2-40+. Irregular mucosal hyperplasia was observed in the lamina propria and submucosa. The inner wall was lined with a single layer of flat epithelium, and the cavity contained red blood cells or lymphatic fluid. Immunohistochemistry was positive for CD34 and/or D2-40 expression, suggesting the presence of proliferative vascular and lymphatic vessels; (**c**) H, HE × 200; (**d**) H, IHC: CD34+; Clustered vascular hyperplasia in the submucosa of the intestine, lumen irregularity, partial dilation, and positive CD34 expression determined by immunohistochemistry all suggested haemangioma; (**e**) L, HE × 200; (**f**) L, IHC: D2-40 + The lymphatic vessels of the lamina propria of the intestinal mucosa showed significant proliferation, and the lumen contained lymphatic fluid. Immunohistochemistry was positive for D2-40 expression.

HL: Irregular mucosal hyperplasia was observed in the lamina propria and submucosa. The inner wall was lined with a single layer of flat epithelium, and the cavity contained red blood cells or lymphatic fluid. Immunohistochemistry was positive for CD34 and/or D2-40 expression, suggesting the presence of proliferative vascular and lymphatic vessels (Fig. 5a,b). H: Clustered vascular hyperplasia in the submucosa of the intestine, lumen irregularity, partial dilation, and positive CD34 expression determined by immunohistochemistry all suggested H (Fig. 5c,d). L: The lymphatic vessels of the lamina propria of the intestinal mucosa showed significant proliferation, and the lumen contained lymphatic fluid. Immunohistochemistry was positive for D2-40 expression (Fig. 5e,f).

### Treatment and prognosis

Of the 31 patients, 3 were followed up without treatment, and 14 had undergone endoscopic resection (EMR/ESD). Among them, 1 patient with duodenal H had undergone endoscopy and had reported the occurrence of bloody stool after discharge, which was cured by medical treatment. Fourteen patients had undergone laparoscopic resection, among whom 1 patient with transverse colonic H had undergone laparoscopic resection and complained of occasional abdominal discomfort postoperatively. One patient with a small intestinal H had undergone laparoscopic resection and showed signs of obstruction recurrence, which was relieved after medical treatment. No significant difference was found between endoscopic resection and laparoscopic resection (P > 0.05) (χ^2^ = 0.373; P = 0.541).

### Follow-up and recurrence

As of 2019-12-29, 3 patients were lost to follow-up, 3 died, and 25 survived. Specifically, 3 patients were lost to follow-up because of the long period; the last visit was used as the end point of follow-up. Of the 3 patients who died, one was treated with laparoscopic surgery for small bowel haemangioma and recovered well. In the second year, he died of underlying diseases, including decompensated liver cirrhosis, primary abdominal bleeding, and hepatorenal syndrome. One of the patients was treated with laparoscopic surgery for small bowel angioma and recovered well after the operation. Later, the patient died of heart disease and heart failure. Additionally, an 80-year-old elderly patient had received EMR treatment for ascending colon L and recovered well after the operation. The patient died because of intestinal cancer lung metastasis.

No significant difference was found in survival, as determined by the log-rank (Mantel-Cox) test (χ^2^ = 2.244, P = 0.326). The survival results for the three pathological types of VTs were consistent. The average follow-up duration among the survivors was 57.06 ± 35.64 months. Except for the 6 who were lost to follow-up and died, the other patients are currently undergoing outpatient follow-up and are still alive. Two people who died had H and one had HL, so the survival rates of the three groups were 100% (8/8) for the HL group, 85.71% (12/14) for the H group, and 88.89% (8/9) for the L group.

## Discussion

VTs have a low incidence and are rarely found clinically. Most reports of VTs in the literature have been case reports^6–10^, with gastrointestinal bleeding as the clinical manifestation. In the present study, an 11-year multicentre retrospective analysis was performed to systematically describe the clinical manifestations, locations, pathological morphology on endoscopy, diagnostic features on imaging, prognosis and survival.

Regarding sex and age, the oldest patient was 84 years, and the youngest was 19 years; both had H. Despite the large age span, most patients were middle-aged, ranging from 44 to 66 years. No difference was found regarding sex in the present study.

Regarding clinical manifestations and location, most patients were asymptomatic, but patients had clinical manifestations such as abdominal pain, nausea and vomiting^11^. In the present study, 32.26% (10/31) of patients had no noticeable symptoms and were identified during endoscopic examination; 45.16% (14/31) were identified because of noticeable clinical manifestations, and bleeding and obstruction occurred in 37.5% (12/31) and 6.45% (2/31) of patients, mainly because of the rapid growth, thin adventitia, and abundant blood supply of VTs. L lesions are benign but can cause serious complications, and they may be related to the abnormal development or inflammation of lymphatic vessels and obstruction of developing lymphatic vessels^12^. Obstruction symptoms appeared only in cases of L, a finding that is also clinically significant and related to the growth pattern of L. Previously, diagnosing gastrointestinal H was difficult, and almost all lesions were confirmed during surgery or by pathology postoperatively^13^; with the introduction of capsule endoscopy (CE) and balloon-assisted enteroscopy (BAE) over the past decades, the small intestine has now become an area that can be targeted^14^. Regarding the location, we found no cases of VT growth in the stomach or oesophagus, which is clinically suggestive. VTs were found in the lower gastrointestinal tract, and mostly small intestinal VTs have been reported in the literature.

Regarding blood indicators, we found that the haemoglobin level in cases of L and HL (lesions with a haemangiomorphology) was significantly higher than that in cases of H, a finding related to the former two being prone to bleeding and causing blood loss. Many case reports have indicated that "anaemia" is a clinical manifestation of VTs^3,7,10,14,15^. All the patients were negative for CEA and AFP, indicating that VTs are benign tumours.

Regarding morphology, no significant difference was found in size among the three types of masses, and we cannot assess the type of pathology by size. In this retrospective study, typical images were provided along with a large amount of endoscopic imaging data, summarized as follows: 1. On endoscopy, most H lesions showed lateral development, a hemispherical shape and a red colour. The outer membrane shows signs of blood filling or even spontaneous bleeding. The surface can show striations or wrapping blood vessels, and the lesion immediately bleeds on biopsy. 2. HL contains L components, is relatively flat in shape or dominated by lateral development and has no special signs in terms of colour. In identification, magnifying endoscopy is particularly advantageous. If large heterogeneous vessels are mixed, the diagnosis of haemangiolymphoma must be considered. The specific colour may be related to the proportion of pathological components. 3. Morphologically, L lesions are similar to VTs and milky white in colour, with some surfaces showing granular structures. If magnifying endoscopy is used to observe white opaque substances and large, heterogeneous blood vessels are found, VTs must be considered instead of simple L and other types of L. These lesions can also present a distinct adventitia, which appears to contain a large amount of lymphatic fluid. This information could serve as a reference for clinical diagnosis.

Pathology provides the final diagnosis of the disease. In all three pathological types, clusters of irregular luminal-like structures in the lamina propria and submucosa can be clearly observed. The inner wall is lined with a single layer of flat epithelium, and the cavity contains red blood cells or lymphatic fluid. Additionally, although the pathological diagnosis can be confirmed by the immunohistochemical expression of CD34 and/or D2-40, in clinical work, for the endoscopic observation of H, pathological biopsy should be used with caution, or direct resection by ESD/EMR/laparoscopic/open surgery should be performed to avoid bleeding on biopsy. VTs are difficult to distinguish on imaging. CT has high clinical value in the diagnosis of H^16^. In the present study, only 31.25% of patients were diagnosed by imaging of the diseased site (5/16), suggesting a lesion. Among them, only 3 patients showed findings suggesting HL. Diagnosing VTs is difficult on CT, which can only indicate multiple speckle-like high-density shadows based on the proportion of vascular components. CT allows a suspected diagnosis of HL or H, but the reliability is not as good as that of endoscopy. Cystic L lesions may be suspected as a cystic disease. It is difficult to identify the components of lymphatic fluid using CT values. According to the European Society of Gastrointestinal Endoscopy (ESGE) guidelines, gastroscopy and balloon-assisted colonoscopy are still recommended for the diagnosis of VTs^17^. In the present study, we also used EUS to examine cystic lesions and found that the hypoechoic shadows originated from the mucosal myometrium and the submucosa, with a final pathological diagnosis of VTs. There are also reports in the literature on the diagnosis of haemorrhagic small intestinal L by EUS, allowing confirmation of the diagnosis by identifying the cystic spaces throughout the submucosa^18^.

Regarding treatment and survival, VTs can be observed during follow-up endoscopically or surgically, depending on the condition. Studies have shown that^13^ endoscopic treatment is suitable for smaller tumours, while laparoscopic/open surgery is recommended for larger tumours. In the present study, 3 patients were not treated and were lost to follow-up, 14 patients had undergone endoscopic resection (EMR/ESD), and 1 patient showed bleeding after EMR and medical treatment. Laparoscopic resection of the lesions was performed routinely in these 14 patients; 3 patients had mild discomfort after laparoscopic resection, which was relieved after symptomatic treatment in all cases. At the same time, 1 case of bleeding was found after biopsy, and the bleeding stopped after medical treatment. During follow-up, there were no cases of recurrence, and 3 patients died of unrelated causes; survival was good for the three pathological types of VTs, with no significant difference. Therefore, these VTs were noninvasive and did not recur. VTs can be treated if clinical symptoms appear and removed if necessary. Follow-up is recommended when no clinical manifestations occur. Blind biopsy or resection should not be performed because it could lead to unpredictable complications and bleeding.

## Conclusion

VTs are rare and can easily be missed in the clinic. VTs are often identified clinically because of bleeding or obstruction and are often cleared pathologically after resection. VTs have clear endoscopic and pathological features, and the prognosis with endoscopy and surgery is good. The disease has no invasive manifestations, with no tendency for recurrence. Clinical symptoms or conditions can be considered to prevent bleeding and obstruction. In most of these cases, the patient can be recommended for clinical follow-up.

## Data Availability

The data used to support the findings of this study are available from the corresponding author upon request.
